# Disparities in Access to Severe Incontinence Surgical Treatment with Artificial Urinary Sphincter after Radical Prostatectomy in France: A Nationwide Nested Case-control Study

**DOI:** 10.1016/j.euros.2026.03.011

**Published:** 2026-04-10

**Authors:** Louis Lenfant, Yoann Taillé, Emmanuel Chartier-Kastler, Bertrand Lukacs, Thomas Seisen, Morgan Rouprêt, Aurélien Beaugerie, Eric Vicaut, Pierre C. Mozer

**Affiliations:** a Sorbonne Université, Service d’urologie, Hôpital Universitaire Pitié-Salpêtrière, AP-HP, Paris, France; b Hôpitaux Saint Louis-Lariboisière-Fernand Widal, AP-HP, Université Paris Cité, Paris, France; c Health Data Hub, AP-HP, Paris France

**Keywords:** Urinary Incontinence, Stress/surgery, Prostatectomy/adverse effects, Urinary Sphincter, Artificial, Health Services Accessibility/statistics and numerical data, Socioeconomic Factors, Disparities/statistics and numerical data

## Abstract

**Background:**

Socioeconomic and geographic disparities in surgical treatment utilization may persist even within universal healthcare systems.

**Objective:**

To determine whether socioeconomic and geographic disparities exist in artificial urinary sphincter (AUS) utilization among men with post-prostatectomy incontinence in a universal healthcare setting.

**Design, setting, and participants:**

This nationwide, population-based, nested case-control study used French administrative health data from 2006 to 2018. Among 718,360 men diagnosed with prostate cancer, 266,927 underwent radical prostatectomy. Of these, 5,064 received AUS for post-prostatectomy incontinence (cases). A comparator group of 3,022 men with documented persistent incontinence but no AUS was identified using repeated penile sheath prescriptions more than one year after surgery.

**Outcome measurements and statistical analysis:**

Associations between AUS implantation and demographic, clinical, socioeconomic, and geographic factors were assessed using multivariable conditional logistic regression accounting for follow-up time and center-level clustering.

**Results and limitations:**

AUS implantation occurred in 1.9% of post-prostatectomy patients. Higher odds of implantation were observed across socioeconomic deprivation categories, with the strongest association in the most socially deprived group (FDEP Q5: OR 1.64, 95% CI 1.32–2.02). Factors linked to lower odds of AUS implantation included older age (70–80 years: OR 0.44, 95% CI 0.36–0.54; >80 years: OR 0.24, 95% CI 0.08–0.78), prior TURP (OR 0.17, 95% CI 0.13–0.22), and radiotherapy (OR 0.73, 95% CI 0.60–0.89). Urban residence was associated with slightly lower odds of AUS implantation compared with rural residence (OR 0.84, 95% CI 0.72–0.99), whereas distance to a high-volume center was not significantly associated.

**Conclusions:**

In France’s universal healthcare system, socioeconomic and geographic factors were not associated with lower odds of receiving AUS among more deprived or rural groups, indicating no evidence of disadvantage for these populations. However, the low overall use of AUS highlights potential non-structural barriers such as limited referral, clinical awareness, or patient engagement that may affect treatment uptake.

## Take Home Message

In a universal health care system, artificial urinary sphincter use after radical prostatectomy does not vary by socioeconomic or geographic factors. However, its very low uptake suggests barriers beyond access—likely related to referral pathways, clinical practice, or patient awareness.

ADVANCING PRACTICE **What does this study add?**

This study demonstrates that, within a universal health care system, there are no significant socioeconomic or geographic disparities in likelihood of receiving artificial urinary sphincter (AUS) implantation among men with postprostatectomy incontinence. By focusing on a rigorously defined control group and accounting for follow-up time, it offers a more precise evaluation of potential inequities in surgical care. Importantly, it reveals that despite equitable structural access, AUS utilization remains low—highlighting the potential influence of nonsystemic barriers such as limited referral, awareness, or patient engagement.

**Clinical Relevance**

These findings suggest that, in France, structural socioeconomic and geographic barriers may not be the main drivers of inequity in access to AUS implantation after radical prostatectomy. However, the very low overall rate of AUS use indicates that other barriers, such as limited referral, insufficient clinical awareness, or low patient engagement, may still substantially restrict treatment uptake. Associate Editor: Véronique Phé.

**Patient Summary**

We studied French men with urinary incontinence after prostate cancer surgery. Although uptake of artificial urinary sphincter surgery was not lower among men living in rural areas or those from more socioeconomically deprived backgrounds, very few men received the standard surgical treatment. This suggests that awareness or referral practices may be limiting treatment.

## 1 Introduction

Stress urinary incontinence (SUI), defined by the International Continence Society as the involuntary loss of urine on effort, physical exertion, or coughing [1], is a common and debilitating complication following radical prostatectomy (RP) [2], [3]. Its severity is typically measured by the frequency of leakage episodes and pad usage. In the ProtecT trial, 36% of men reported pad use 1 yr after RP, decreasing to 20% at 6 yr [4]. Furthermore, findings from Joshua Bridge et al indicate that 1 yr after RP, 5% of patients used two pads per day, and 4% used more than two pads per day, underscoring the significant burden of severe incontinence after prostate cancer (PCa) radical surgical treatments [5].

The artificial urinary sphincter (AUS) remains the gold standard for treating severe SUI [6], [7], although it may also be considered in selected cases of moderate, and occasionally mild, incontinence when alternative treatments are inappropriate or ineffective. However, population-based studies show that only 2–4% of men undergo incontinence surgery after RP [8], [9], suggesting a substantial gap between the prevalence of severe incontinence and the use of surgical management [10], [11].

Prior research in the United States, using Medicare or State Inpatient databases, have highlighted substantial socioeconomic disparities in access to surgical care for postprostatectomy complications [9], [12]. However, their design included all men after RP—both continent and incontinent—as controls. Such an approach conflates the likelihood of developing incontinence with the likelihood of receiving surgical treatment, thereby limiting the ability to isolate factors specifically influencing access to care among incontinent patients. Moreover, the exclusion of younger men and the absence of outpatient data further restrict generalizability.

France offers a unique context for investigating such disparities within a universal health care system. Using nationwide data, this study aimed to primarily assess socioeconomic and geographic disparities in AUS utilization among men with postprostatectomy incontinence. A secondary objective is to identify clinical and demographic predictors associated with AUS use. By distinguishing between inequities and clinical determinants, this study aims to inform policies that promote equitable availability and use of surgical care for postprostatectomy incontinence.

## 2 Materials and Methods

### 2.1 Study design

This study was a nested case-control analysis within a national population-based cohort using comprehensive French health care databases. We used patient-level data from the ObservaPUR database, which integrates information from the French Hospital Discharge Database (Programme de Médicalisation des Systèmes d’Information [PMSI]) and the National Health Insurance Information System (Système National Inter-Régimes de l’Assurance Maladie [SNIIRAM]). These databases provide nationwide coverage, recording all inpatient hospital stays, outpatient care, and reimbursed medications across France. Ethical approval for this study was granted by the French National Commission for Data Protection and Liberties (CNIL; approval number DE-2010-002, July 2010), ensuring compliance with European data protection regulations. Patients or the public were not involved in the design, or conduct, or reporting, or dissemination plans of our research.

### 2.2 Population

We identified all patients treated for PCa in France between January 1, 2006, and December 31, 2018. The *intervention cohort* consisted of patients who underwent their first elective peri-bulbar AUS implantation after RP, with no minimum or maximum interval required between RP and AUS implantation. Early implantations are rare in practice as AUS is generally deferred for several months post-RP. AUS procedures were identified using the French Common Classification of Medical Procedures [13] (Classification Commune des Actes Médicaux [CCAM]) code JELA002 and the specific medical device code from the List of Reimbursable Products and Services (Liste des Produits et Prestations [LPP]) LPP : 3121402. The control cohort included patients without AUS implantation who had severe postprostatectomy urinary incontinence, identified by the prescription of penile sheaths more than 1 yr post-RP with at least 1 mo of continuous prescription. To ensure complete follow-up and avoid differential observation time, we restricted RP inclusion to January 1, 2006 to November 30, 2017, providing at least 12 mo of follow-up before the administrative censoring date (December 31, 2018). No maximum interval between RP and the first penile sheath prescription was imposed. Penile sheaths prescriptions were identified using specific LPP codes listed in the Supplementary Material (Supplementary 1). These reimbursed devices are provided for documented urinary leakage, offering an objective indicator of persistent postprostatectomy incontinence.

### 2.3 Data collection

The PMSI database provided patient-level information, including demographics (age, sex), details of hospital admissions (admission and discharge dates), and clinical data (diagnoses, comorbidities, surgical procedures, and medical devices used). The PMSI database provided information on patients’ residential locations, allowing assessment of area-based socioeconomic deprivation using the French Deprivation Index (FDEP) score [14]. The index, derived from data provided by the French National Institute of Statistics and Economic Studies (Institut National de la Statistique et des Études Économiques [INSEE]), incorporates metrics such as unemployment rate, proportion of manual workers, education level, and median income. Patients were categorized as less deprived (lowest quintiles) or more deprived (highest quintiles) based on their FDEP score. In the PMSI, diagnoses are coded according to the International Classification of Diseases and Related Health Problems, Tenth Revision (ICD-10), [15]. Surgical procedures are classified using the CCAM classification [13], and implanted medical devices are systematically coded using a specific List of Reimbursable Products and Services (with a “LPP” code). Previous research has demonstrated that the French National Health Data System (Système National des Données de Santé [SNDS]), which includes hospital discharge data (PMSI), is sufficiently robust for research and can support public health decision-making [16].

Patients’ residential locations were represented by the geographic coordinates of their municipalities as recorded in the PMSI. Residential locations were categorized as rural or urban following the INSEE classification of urban units in 2020 [17].

The data extracted from the SNIIRAM database, to constitute the ObservaPUR database, included all prescriptions related to treatments for diabetes, hyper-tension, androgen deprivation therapy, GnRH inhibitors, as well as anticoagulation, antiplatelet, and anti-muscarinic therapies.

### 2.4 Statistical analysis

Baseline characteristics of patients with postprostatectomy incontinence were summarized using medians and interquartile ranges for continuous variables and counts with percentages for categorical variables. Comparisons between patients who received an AUS and those who did not were performed using the Wilcoxon rank-sum test for continuous variables and Pearson’s chi-squared test for categorical variables, as presented in Table 1, Table 2.

**Table 1:** Baseline characteristics of postprostatectomy incontinent patients in France between 2006 and 2018

**Characteristics**	**Control group** **Incontinent after RP without AUS** *N =* 3022^a^	**Intervention group** **Incontinent after RP with AUS** *N =* 5064^a^	***p* value**^b^
**Age at prostate surgery (IQR)**	67 (63–71)	66 (61–70)	**<0.001**
**Prostate cancer surgical treatment *- n* (%)**			**<0.001**
No prostate cancer surgery	510 (17%)	12 (0.2%)	
Ablatherm	18 (0.6%)	1 (<0.1%)	
Brachytherapy	5 (0.2%)	0 (0%)	
Laparoscopic radical prostatectomy	1075 (36%)	2558 (51%)	
Open radical prostatectomy	1411 (47%)	2481 (49%)	
Perineal radical prostatectomy	6 (0.2%)	12 (0.2%)	
**Radical prostatectomy and radiation therapy - *n* (%)**	398 (13%)	535 (11%)	**0.001**
**BPH surgical treatment - *n* (%)**			**<0.001**
No BPH surgery	2241 (74%)	4691 (93%)	
Bladder neck incision	0 (0%)	13 (0.3%)	
Laser endoscopic surgery	6 (0.2%)	0 (0%)	
Simple prostatectomy	88 (2.9%)	93 (1.8%)	
Transurethral needle ablation	3 (<0.1%)	6 (0.1%)	
TURP	684 (23%)	261 (5.2%)	
**Urethra or bladder neck surgery - *n* (%)**			**<0.001**
No prior urethra or bladder neck surgery	2402 (79%)	4423 (87%)	
Bladder neck incision	0 (0%)	0 (0%)	
Urethral stenosis surgery	534 (18%)	577 (11%)	
Other	86 (2.8%)	64 (1.3%)	

Bold values refers ***p***<0.05.

AUS *=* artificial urinary sphincter; BPH *=* benign prostatic hyperplasia; IQR *=* interquartile range; TURP *=* trans urethral resection of the prostate.

a Median (IQR); n (%).

b Wilcoxon rank sum test; Pearson’s chi-squared test.

**Table 2:** Baseline comorbidities, socio-economic and geographic characteristics of postprostatectomy incontinent patients in France between 2006 and 2018

**Characteristics**	**Control group** **Incontinent after RP without AUS** *N =* 3022^a^	**Intervention group** **Incontinent after RP with AUS** *N =* 5064^a^	***p* value**^b^
**Diabetes - *n* (%)**	664 (22%)	1051 (21%)	0.2
**Obesity- *n* (%)**	522 (17%)	1011 (20%)	**0.003**
**Tobacco use - *n* (%)**	451 (15%)	588 (12%)	**<0.001**
**French Deprivation Index (FDep09) quintiles - *n* (%)**			**<0.001**
Q1	617 (23%)	881 (18%)	
Q2	518 (19%)	986 (21%)	
Q3	540 (20%)	964 (20%)	
Q4	555 (20%)	954 (20%)	
Q5	501 (18%)	1002 (21%)	
Missing	291	277	
**Rural localization - *n* (%)**	625 (24%)	1232 (26%)	0.08
Missing	417	320	
**Distance to nearest expert center, km (IQR)**	27 (9–57)	30 (10–59)	**0.015**

Bold values refers ***p***<0.05.

a Median (IQR); n (%).

b Wilcoxon rank sum test; Pearson’s chi-squared test.

We fitted a multivariable conditional logistic regression model to estimate the association between patient characteristics and AUS implantation. Because AUS implantation is an inherently time-dependent event and follow-up duration may vary between patients, the primary analysis was conducted using a nested case-control design based on incidence density sampling. For each patient receiving AUS, a risk set was defined consisting of patients who had not undergone AUS and who were still under observation at the time of the case’s implantation, thereby ensuring comparable opportunity for treatment. Controls were sampled from the eligible pool with replacement, meaning that the same individual could be selected as a control for multiple case sets. In our implementation, control eligibility required no AUS during follow-up; therefore, individuals who later received an AUS were not eligible to serve as controls and could not become cases after being selected as controls. Up to five controls were randomly selected from each risk set, and associations were estimated by conditional logistic regression with the Efron approximation to obtain valid clustered standard errors, stratified by matched risk sets. Under this sampling scheme, odds ratios (ORs) can be interpreted as estimates of hazard ratios.

Clustering by the center where RP was performed was accounted for by using robust standard errors clustered at the center level, identified using the FINESS institutional code (562 centers), thereby capturing shared referral pathways, local practice patterns, and center-level differences in surgical practice. Temporal trends were addressed by modeling the calendar year of RP as a continuous centered variable, in accordance with contemporary statistical guidelines [18]. All remaining covariates were included as fixed effects, and the dependent variable was AUS implantation.

The model included the following predictors, defined a priori. Age at RP was categorized into four groups (<60, 60–70, 70–80, and >80 yr). Benign prostatic hyperplasia (BPH) surgery was classified as none, transurethral resection of the prostate (TURP), simple prostatectomy, or other procedures. Radiotherapy, obesity, diabetes, and tobacco use were entered as binary variables (yes/no). Social deprivation was measured using the FDEP index and included as quintiles (Q1–Q5). Geographic covariates comprised commune type (urban or rural) and distance to the nearest high-volume AUS center (≤100 vs >100 km). Multicollinearity was assessed using variance inflation factors (VIF) and bivariate associations. All VIF values were <1.1, indicating no evidence of problematic collinearity among the socioeconomic and geographic variables in the fitted model. Results are reported as ORs with 95% CIs. The multivariable analysis was performed as a complete case analysis, including only patients with complete data for all covariates entered in the model.

A *p* value <0.05 was considered statistically significant. Statistical analyses were performed using the R statistical software, version 4.2.2 (Vienna, Austria).

## 3 Results

**Fig. 1:**
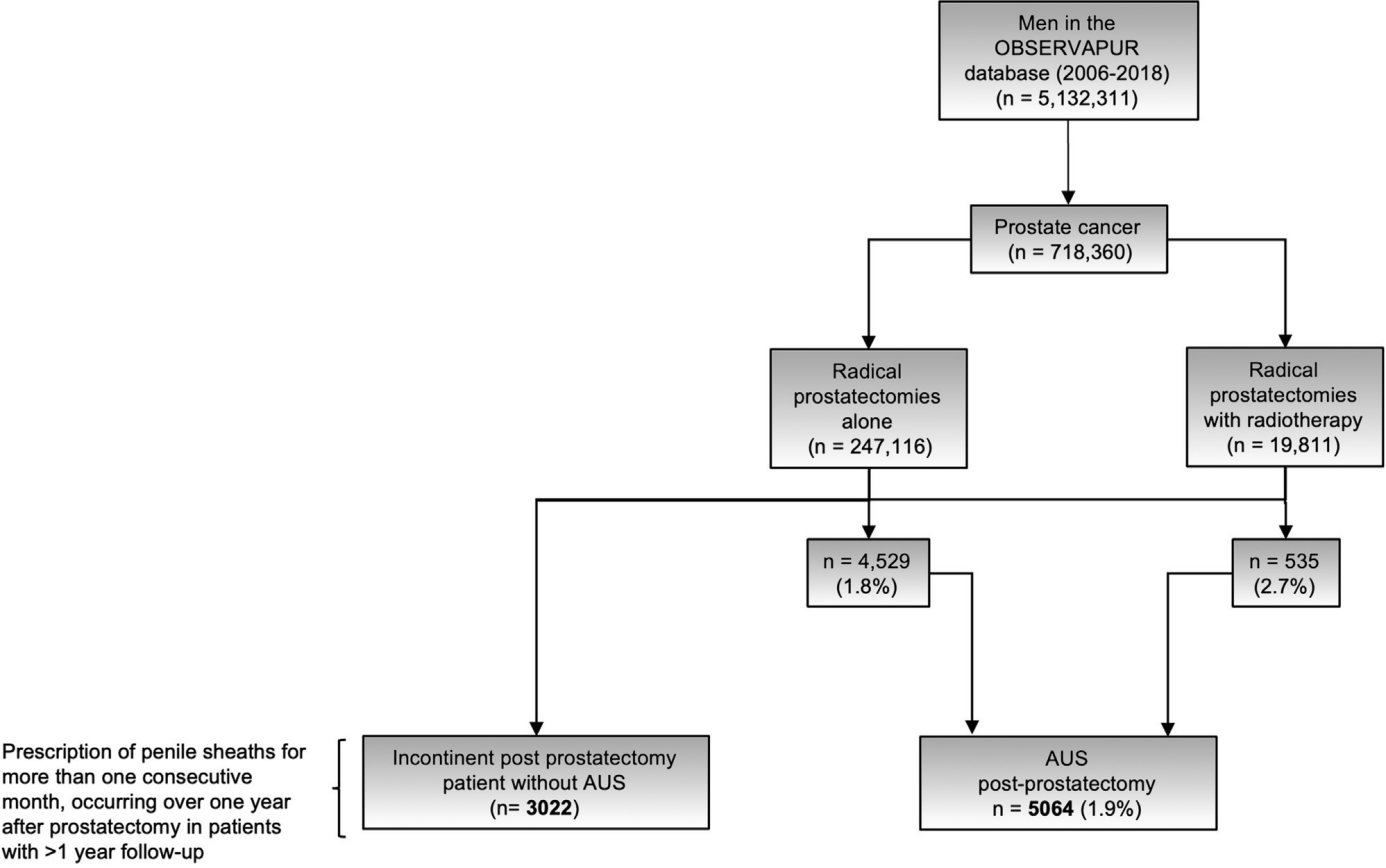
**Flowchart of the study population selection.** This diagram illustrates the identification process of men with postprostatectomy incontinence from the ObservaPUR database (2006–2018). From over 5 million male patients, those with prostate cancer and subsequent radical prostatectomy (with or without radiotherapy) were identified. Postoperative urinary incontinence was defined based on penile sheath (LPP) prescriptions lasting more than one consecutive month and occurring more than 1 yr after surgery, in patients without a history of artificial urinary sphincter (AUS). The final analytic sample included 3090 incontinent postprostatectomy patients without AUS, and 5064 patients with AUS implantation. AUS *=* artificial urinary sphincter; LPP *=* Liste des Produits et Prestations (list of reimbursable products and services).

Out of the 5 132 311 men in the ObservaPUR database, 718 360 were treated for PCa. Among these, 247 116 underwent RP alone, and 19 811 underwent RP combined with radiotherapy (RT). In total, 5064 men underwent AUS implantation post-RP, accounting for 1.9% (5064/266 927) of the intervention cohort. Specifically, 4529 (1.8%) of men with RP alone and 535 (2.7%) of men with RP combined with RT received AUS. The control cohort consisted of 3022 men who had a prescription for a penile sheath for more than 1 mo more than 1 yr after surgery, with no prior history of AUS implantation (Fig. 1). Among men with documented persistent postprostatectomy incontinence—identified either through AUS implantation or repeated penile sheath prescriptions—63% received an AUS.

### 3.1 Patient characteristics

The characteristics of the study population are presented in Table 1. Prior BPH surgery was significantly less frequent in the intervention group compared to the control group, with no prior BPH surgery reported in 93% versus 74% (*p <* 0.001). TURP was more frequent in the control group (5.2% vs 23%, *p <* 0.001).

Regarding PCa surgical treatment, laparoscopic RP was more frequently performed in the intervention group (51% vs 36%, *p <* 0.001), while the control group had a slightly higher proportion of open RP (47% vs 49%). RT was slightly more frequent in the control group (11% vs13%, *p <* 0.001).

Prior urethral or bladder neck surgery also differed significantly between groups. Urethral stenosis surgery was more prevalent in the control group (11% vs 17%, *p <* 0.001).

### 3.2 Comorbidities

No significant difference was observed in the prevalence of diabetes between groups (*p =* 0.2). However, obesity was more common in the intervention group (20% vs 17%, *p =* 0.003). Tobacco use was slightly higher in the control group (12% vs 15%, *p <* 0.001).

### 3.3 Socioeconomic and geographic factors

The FDEP index indicated slightly higher social deprivation in the intervention group (median 0.3 vs 0.2, *p <* 0.001). Urban localization was predominant across both cohorts, with no significant difference between groups (*p =* 0.08). Median distance to a high-volume center (≥20 AUS/yr) differed between groups (30 vs 27 km, *p =* 0.015), although this difference is unlikely to be clinically meaningful.

### 3.4 Factors associated with AUS implantation

In multivariable analysis, older age was strongly associated with lower odds of AUS implantation, particularly among men aged 70–80 yr (OR 0.44, 95% CI 0.36–0.54) and >80 yr (OR 0.24, 95% CI 0.08–0.78). Prior TURP (OR 0.17, 95% CI 0.13–0.22) and radiation therapy (OR 0.73, 95% CI 0.60–0.89) were also associated with lower odds of AUS implantation.

**Fig. 2:**
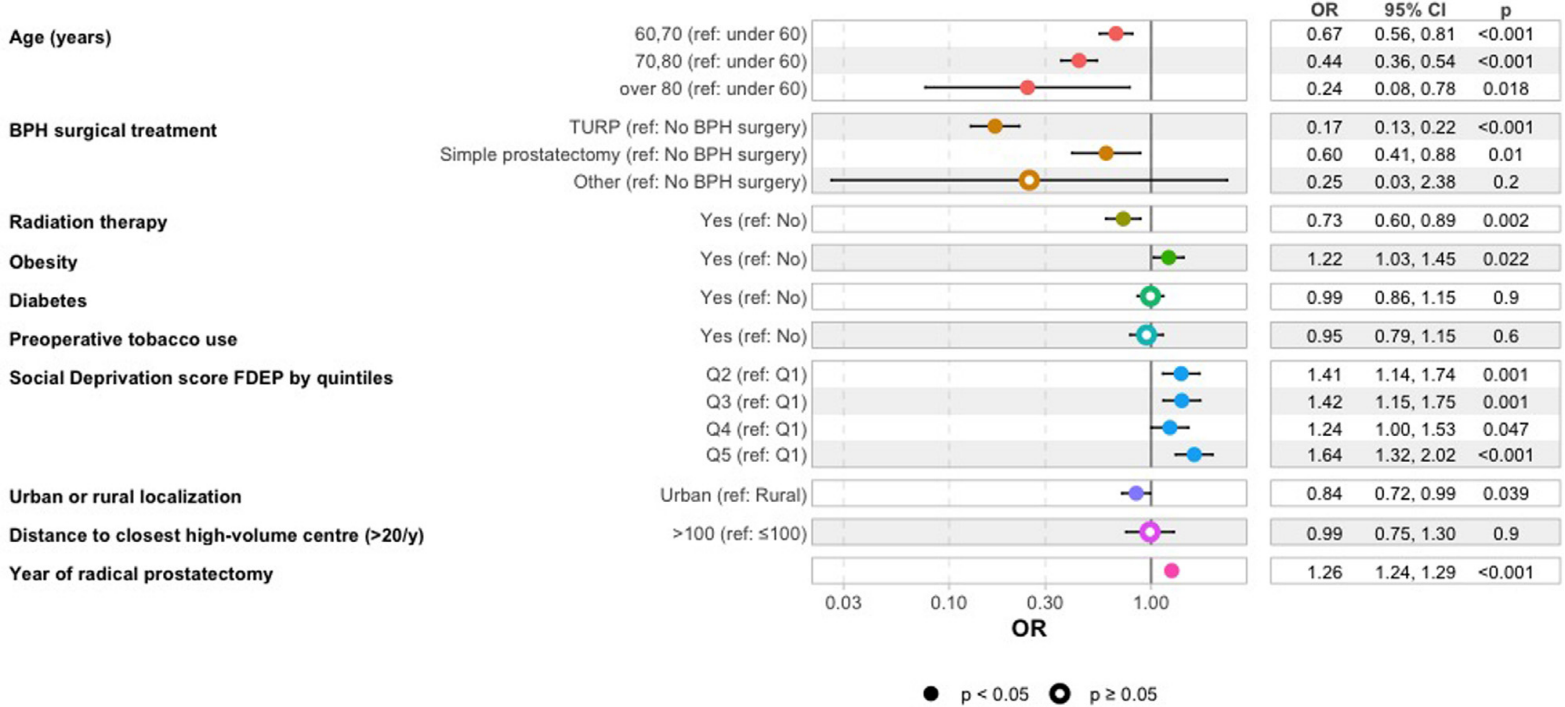
**Multivariable logistic regression analysis of factors associated with AUS implantation after prostatectomy**. Odds ratios (ORs) and 95% confidence intervals (CIs) for variables associated with AUS implantation are presented. Significant factors include age, prior BPH surgery type (TURP and simple prostatectomy), radiation therapy, obesity, tobacco use, and socioeconomic deprivation (FDEP quintiles). Urban versus rural location and distance to a high-volume center (>100 km vs ≤100 km) were not significantly associated. Markers indicate significance level: solid dots for *p* < 0.05, hollow dots for *p* ≥ 0.05. AUS *=* artificial urinary sphincter; BPH *=* benign prostatic hyperplasia; CI *=* confidence interval; FDEP *=* French Deprivation Index; OR *=* odds ratio; ref *=* reference category.

Conversely, patients in the most socially deprived group (FDEP Q5) had significantly higher odds of AUS implantation (OR 1.64, 95% CI 1.32–2.02). Higher odds were also observed for Q2 (OR 1.41, 95% CI 1.14–1.74) and Q3 (OR 1.42, 95% CI 1.15–1.75), while the association for Q4 was of smaller magnitude but remained statistically significant (OR 1.24, 95% CI 1.00–1.53). Urban residence was associated with slightly lower odds of AUS implantation compared with rural residence (OR 0.84, 95% CI 0.72–0.99), whereas distance to a high-volume center was not significantly associated with AUS utilization (OR 0.99, 95% CI 0.75–1.30; Fig. 2). The year of RP was positively associated with AUS implantation, with more recent surgery years showing slightly higher odds of receiving an AUS (OR 1.26/yr, 95% CI 1.24–1.29).

## 4 Discussion

Our study provides new insights into the utilization of AUS implantation among patients with severe incontinence following RP in France. Unlike studies conducted in other health care systems, particularly the United States [9], [12], our findings reveal no evidence that socioeconomic status or distance to a high-volume center reduced the likelihood of receiving AUS. This is a notable finding, highlighting the potential benefits of a universal health care system in mitigating inequities in access to specialized surgical treatments.

A significant strength of our study is the inclusion of a control cohort of incontinent patients who did not undergo surgical treatment, allowing us to identify prognostic factors for AUS implantation when indicated. Previous studies aimed to identify predictive factors for incontinence surgery, but their control groups included patients who did not undergo surgery [9], [12]. These control populations consisted of continent patients who did not require incontinence surgery and incontinent patients who did not have access to surgery. As a result, the predictive factors identified in these studies reflected both the occurrence of incontinence and the access to incontinence surgery. By defining a control group composed solely of incontinent patients who did not receive AUS surgery, our study seeks to more precisely identify the factors that decrease the odds of receiving AUS surgery when it is indicated. By selecting control group patients based on the use of penile sheaths, we ensured the inclusion of individuals with objectively severe incontinence. In France, the cost of pads is not covered by the national health insurance, making their use neither measurable nor identifiable in health databases. However, patients with severe incontinence often alternate between pads and penile sheaths depending on circumstances and activities [19]. Our selection criteria therefore enable the identification of individuals with persistent and severe incontinence 1 yr after prostatectomy.

Our results showed that age, prior radiation therapy (RT), and prior BPH surgery were associated with decreased odds of AUS implantation. Men aged ≥60–70 yr had progressively lower odds of receiving AUS compared to younger men, consistent with findings from Nelson et al, who reported that older age and comorbidities were predictors of incontinence surgery [9]. Similarly, prior RT was a significant negative predictor, likely due to the increased complexity of surgery in this patient subgroup, as documented in the literature [20], [21].

Importantly, our study found no evidence that socioeconomic factors limited the likelihood of receiving AUS. In contrast, men from more socioeconomically deprived areas consistently showed higher odds of implantation across deprivation quintiles, suggesting differences in utilization rather than restricted access. This contrasts with studies from the United States, such as those by Adesanya et al and Gupta et al, which highlighted significant racial and income-based disparities in access to incontinence surgery [12], [22]. Adesanya et al found that White men were more likely to receive surgical treatment than Black men, with income levels also influencing access [12]. Similarly, Gupta et al demonstrated a marked underutilization of AUS and sling procedures among Black patients compared to White patients [22]. In another analysis, McAbee et al reported that most AUS and male sling procedures were performed on White men (87.2%) compared to Black men (9%), further emphasizing the racial disparities in surgical care [23]. Interestingly, in the present study, men from more deprived areas had consistently higher odds of AUS implantation. This paradoxical finding may reflect the greater availability of follow-up and specialized care in public university hospitals, which are fully covered without additional costs, and therefore, more frequently used by socioeconomically disadvantaged patients. However, as the FDEP is an area-based index derived from residential zip codes, ecological bias cannot be excluded, and residual referral or coding effects may also contribute to this trend. Urban residence was associated with slightly lower odds of AUS implantation compared with rural residence, although the magnitude of this effect was modest and may reflect differences in care pathways rather than true geographic barriers.

The absence of such disparities in our study likely reflects the influence of France’s universal health care system, which provides equitable availability and use of surgical interventions. The structural design of the French system minimizes financial barriers and geographic inequalities—challenges that are prominent in the United States (US) health care model. This finding is consistent with the core principles of universal health systems, where access is not contingent on individual socioeconomic status, but is instead guaranteed based on clinical need. According to Oliver and Mossialos [24], such systems are designed to promote horizontal equity—that is, equal access for equal need—a principle clearly reflected in our results. Furthermore, our study did not evaluate racial disparities, as such, data collection is prohibited by the first article of the French Constitution. While this limitation prevents direct racial comparisons, the absence of socioeconomic disadvantage in access, and the lack of major geographic barriers, reinforce the equity-driven approach of France’s health care system.

Despite the lack of disparities, our study highlights the relatively low overall utilization of AUS implantation, with only 1.9% of post-RP patients undergoing the procedure. This is consistent with findings from Nelson et al, who documented a significant gap between the prevalence of bothersome incontinence and the rate of surgical correction [9]. This gap raises concerns about barriers unrelated to socioeconomic or geographic factors, such as patient reluctance to seek care, embarrassment, or inadequate follow-up. Similar patterns were observed in the TrueNTH Post Surgery UK cohort, where one in three men continued to wear pads and nearly half reported urinary leakage 12 mo after RP; however, only 10% described it as a moderate or big problem [5]. These findings suggest that underreporting of symptom burden, low patient expectations of recovery, or limited awareness of available treatments may contribute to the underutilization of surgical solutions like AUS, even in publicly funded health systems. These barriers, as described in prior US-based studies, may also be present in France but are less likely to result from financial or systemic inequities. Future research should investigate nonsystemic barriers to care, such as cultural perceptions, patient–provider communication, and postoperative expectations.

Our study is not devoid of limitations. First, the lack of data on racial or ethnic disparities due to constitutional restrictions in France limits our ability to compare findings with US-based studies where racial disparities are a significant focus. Second, although our analysis identified predictors of AUS utilization, it did not include patient-reported outcomes, which are essential to capture the functional and psychological burden of incontinence and patients’ decision-making processes. Administrative data provide robust coverage at the population level but cannot account for subjective symptom severity, quality of life, or patient preferences, all of which likely influence treatment uptake. Similarly, because continence severity is not available in the SNDS, some residual differences in baseline incontinence severity between AUS recipients and controls cannot be excluded, even though AUS may occasionally be offered for selected moderate or mild cases. Future studies should therefore consider linking national health care data with clinical and patient-reported datasets to better contextualize care pathways and improve understanding of barriers to surgical treatment. Third, although we addressed differential follow-up using a risk-set matched design, some residual asymmetry in observation and documentation requirements between cases and controls may persist. Control classification requires survival, continued follow-up, and documented incontinence-related prescriptions beyond 12 mo after prostatectomy, whereas cases are identified at the time of AUS implantation. While delayed implantation of AUS likely mitigates this issue, survivor or ascertainment bias cannot be entirely excluded. Fourth, this study relied on administrative coding for diagnoses and procedures, which may introduce misclassification despite rigorous validation of French hospital and insurance databases. Nevertheless, coding errors are unlikely to differ systematically between cases and controls and should not have biased associations directionally. Fifth, the identification of incontinent patients based on penile sheath prescriptions, while innovative and specific, may not capture all men with severe incontinence. In France, penile sheaths are reimbursed only for documented urinary leakage, making them a reliable indicator of persistent incontinence. However, some patients may rely exclusively on nonreimbursed absorbent pads or may have discontinued care due to adaptation, discouragement, or lack of follow-up. As a result, this approach may underestimate the true prevalence of severe incontinence. Nevertheless, any potential misclassification is likely nondifferential between groups and thus would tend to attenuate, rather than inflate, observed associations. Sixth, the study period ended in 2018, and more recent changes in surgical practice may not be fully captured, representing a temporal limitation. Lastly, while France’s universal health care model promotes equity, the generalizability of our findings to countries with different health care systems may be limited.

## 5 Conclusion

In this nationwide analysis, we did not observe evidence of socioeconomic or geographic disadvantage in AUS utilization, underscoring the strengths of a universal health care system in promoting equitable availability and use of specialized surgical care. However, the very low overall utilization of AUS—despite its established role as the standard treatment for severe postprostatectomy incontinence—suggests that barriers other than structural inequalities are likely limiting the use of AUS.

These findings imply that efforts to improve the use of AUS should focus not on reducing disparities but on enhancing general availability and uptake, for example by improving postoperative follow-up, strengthening referral pathways, and increasing patient and clinician awareness of treatment options. Future research should explore patient-level, behavioral, and organizational factors that may influence decision-making and contribute to the underuse of AUS, even in an equitable health care environment.

  ***Authors contributions:*** Louis Lenfant had full access to all the study data and takes responsibility for integrity of the data and the accuracy of the data analysis.

  *Concept and design*: Lenfant, Mozer, Vicaut.

*Acquisition, analysis, and interpretation of data*: Lenfant, Taillé, Vicaut, Lukacs, Mozer.

*Drafting of the manuscript*: Lenfant.

*Critical review of the manuscript*: Vicaut, Mozer, Chartier-Kastler, Rouprêt, Beaugerie, Seisen.

*Statistical analysis*: Lenfant.

*Obtain funding*: None.

*Administrative, technical, or material support*: None.

*Supervision*: Mozer, Vicaut, Chartier-Kastler.

  ***Financial disclosures:*** Louis Lenfant certifies that all conflicts of interest, including specific financial interests and relationships and affiliations relevant to the subject matter or materials discussed in the manuscript (eg, employment/affiliation, grants or funding, consultancies, honoraria, stock ownership or options, expert testimony, royalties, or patents filed, received, or pending), are the following: Chartier-Kastler is a consultant for Boston Scientific, a consultant and speaker for UroMems, a consultant and/or investigator and/or speaker for Coloplast, BBraun, Convatec, as well as a consultant and speaker for Medtronic. Mozer is a consultant and shareholder of the Uromems Company. Beaugerie is a consultant for the Uromems Company. Rouprêt is a consultant and/or speaker for Arquer Diagnostics, Cepheid, Merck Sharp & Dohme, Nucleix, and Roche. Other authors have nothing to disclose.

  ***Funding/Support and role of the sponsor:*** This ObservaPUR project was financed by the Clinical Research Unit, Lariboisière Fernand Widal Hospital, Assistance Publique – Hôpitaux de Paris, University Denis Diderot, Paris, France. A grant was received from the “Association Nationale des Malades du Cancer de la Prostate”.

This academic study was designed, conducted, analyzed, and written completely independently of any sponsor. The “Association Nationale des Malades du Cancer de la Prostate” did not participate in the design or conduct of the study; the collection, analysis, and interpretation of the data; or the preparation, review, or approval of the manuscript.

  ***Data sharing statement:*** Some aggregated data for this study could be shared upon reasonable request to the corresponding author and after agreement of the ObservaPUR Board and the CNAM.
